# Predictors of remission in depression to individual and combined treatments (PReDICT): study protocol for a randomized controlled trial

**DOI:** 10.1186/1745-6215-13-106

**Published:** 2012-07-09

**Authors:** Boadie W Dunlop, Elisabeth B Binder, Joseph F Cubells, Mark M Goodman, Mary E Kelley, Becky Kinkead, Michael Kutner, Charles B Nemeroff, D Jeffrey Newport, Michael J Owens, Thaddeus W W Pace, James C Ritchie, Vivianne Aponte Rivera, Drew Westen, W Edward Craighead, Helen S Mayberg

**Affiliations:** 1 Department of Psychiatry and Behavioral Sciences, Emory University School of Medicine, 1256 Briarcliff Road, Building A, 3rd Floor, Atlanta, GA 30306, USA; 2 Max Planck Institute of Psychiatry, Munich, Germany; 3 Department of Biostatistics and Bioinformatics, Rollins School of Public Health, Emory University, Atlanta, GA, USA; 4 Department of Psychiatry and Behavioral Sciences, University of Miami Miller School of Medicine, Miami, FL, USA; 5 Department of Clinical Pathology, Emory University School of Medicine, Atlanta, GA, USA; 6 Department of Radiology and Imaging Sciences, Emory University School of Medicine, Atlanta, GA, USA; 7 Department of Psychology, Emory University, Atlanta, GA, USA

**Keywords:** Antidepressive agents, Clinical research protocol, Cognitive behavior therapy, Depression, Genetic polymorphisms, HPA Axis, Inflammation, Magnetic resonance imaging, Personality disorders, Personalized medicine

## Abstract

**Background:**

Limited controlled data exist to guide treatment choices for clinicians caring for patients with major depressive disorder (MDD). Although many putative predictors of treatment response have been reported, most were identified through retrospective analyses of existing datasets and very few have been replicated in a manner that can impact clinical practice. One major confound in previous studies examining predictors of treatment response is the patient’s treatment history, which may affect both the predictor of interest and treatment outcomes. Moreover, prior treatment history provides an important source of selection bias, thereby limiting generalizability. Consequently, we initiated a randomized clinical trial designed to identify factors that moderate response to three treatments for MDD among patients never treated previously for the condition.

**Methods/design:**

Treatment-naïve adults aged 18 to 65 years with moderate-to-severe, non-psychotic MDD are randomized equally to one of three 12-week treatment arms: (1) cognitive behavior therapy (CBT, 16 sessions); (2) duloxetine (30–60 mg/d); or (3) escitalopram (10–20 mg/d). Prior to randomization, patients undergo multiple assessments, including resting state functional magnetic resonance imaging (fMRI), immune markers, DNA and gene expression products, and dexamethasone-corticotropin-releasing hormone (Dex/CRH) testing. Prior to or shortly after randomization, patients also complete a comprehensive personality assessment. Repeat assessment of the biological measures (fMRI, immune markers, and gene expression products) occurs at an early time-point in treatment, and upon completion of 12-week treatment, when a second Dex/CRH test is also conducted. Patients remitting by the end of this acute treatment phase are then eligible to enter a 21-month follow-up phase, with quarterly visits to monitor for recurrence. Non-remitters are offered augmentation treatment for a second 12-week course of treatment, during which they receive a combination of CBT and antidepressant medication. Predictors of the primary outcome, remission, will be identified for overall and treatment-specific effects, and a statistical model incorporating multiple predictors will be developed to predict outcomes.

**Discussion:**

The PReDICT study’s evaluation of biological, psychological, and clinical factors that may differentially impact treatment outcomes represents a sizeable step toward developing personalized treatments for MDD. Identified predictors should help guide the selection of initial treatments, and identify those patients most vulnerable to recurrence, who thus warrant maintenance or combination treatments to achieve and maintain wellness.

**Trial registration:**

Clinicaltrials.gov Identifier: NCT00360399. Registered 02 AUG 2006. First patient randomized 09 FEB 2007.

## Background

Major depressive disorder (MDD) is a common, frequently chronic, disabling, and debilitating psychiatric disorder. Approximately 17% of US citizens will at some point in their lives experience MDD with roughly twice as many females as males suffering from the disorder [1]. A widely cited study, Global Burden of Disease, a collaboration of the World Bank, the World Health Organization, and the Harvard School of Public Health, reported that MDD was the fourth most disabling disorder, and predicted that by 2020 MDD would be the second leading cause of disability worldwide, trailing only coronary artery disease [2]. Although it is difficult to ascertain the financial burden of MDD, one well-conducted study in the US found that among primary care patients, healthcare costs of individuals suffering from MDD were twice those of individuals without MDD [3]; these differences resulted largely from increased healthcare costs associated with depressed patients’ utilization of medical services at four times the rate of patients who did not suffer from depression.

The initial onset of MDD occurs most frequently between the ages of 15 years and 29 years, and recent studies have found that approximately 50% of teenagers who are diagnosed with MDD experience a second episode by the age of 25 years [4,5]. One particularly problematic aspect of MDD is the increased likelihood for recurrence following each successive episode [6] with an ultimate mean of approximately 5 years between episodes [7]. Furthermore, MDD is associated with an increased risk of medical disorders, including cardiovascular and endocrine diseases [8-10]. In sum, MDD frequently begins during the teenage and early adult years, and it continues over the life span causing substantial negative social, economic, and health effects [11]. The past few decades have witnessed the development and evaluation of new antidepressant medications and psychotherapies for MDD [12,13]. Despite these advances, depression treatment continues to be hampered by two major limitations: an unacceptably low rate of symptomatic remission, and the virtual absence of any practical predictors of treatment response, whether partial or complete. Although it is widely considered that current interventions benefit approximately 60% of MDD patients, only about 30% to 40% of patients show full remission of their symptoms as defined by the MacArthur criteria (for example, a 17-item Hamilton Depression Rating Scale (HDRS) score <8) [14,15]. Approximately 30% more demonstrate some response to treatment, indicating they experience clinically important reductions in their depressive symptom burden, yet also continue to experience clinically important residual symptoms. Patients who experience a response short of remission are more vulnerable to relapses and recurrences of the disorder [16] and experience greater functional disability [17].

Current treatment for MDD involves a trial-and-error approach because there are no consistently identified predictors of differential response across treatment modalities. Primary first-line treatments consist of either an evidence-based form of psychotherapy or antidepressant medication. In patients treated pharmacologically, several treatment options are available, but there are very few clinical trial data to guide clinicians as to which first step enhances the odds of remission for an individual patient [18]. Illustrating the importance of such trials, one retrospective study found a differential advantage for psychotherapy over medication (nefazodone) in chronically depressed patients with early life trauma [19]. A treatment regimen that ultimately proves to be ineffective results in continuing patient distress and role dysfunction, discouragement regarding possible relief from MDD, exposure to potential side effects, and unnecessary medical costs. Among the roughly 70% of depressed patients who do not remit with their first treatment, many do not return to explore other treatment options that might have proven effective [20].

Previous efforts attempting to identify predictors of treatment response have typically been *post-hoc* analyses of datasets designed to test other hypotheses [18]. Numerous predictors have been identified by this approach, but they lack consistent prospective validation, and such predictive studies generally examine a single treatment. Examples of potential demographic predictors of treatment response have included age, gender, marital status, family history of treatment response, and socioeconomic factors [15]. Clinical predictors have included diagnostic subtype [21,22], severity of depression [23], chronicity [15], symptom profiles [24], patient treatment preference [25], early life stress [19], personality profiles [26], previous treatment [27], psychomotor speed [28], and co-morbid diagnoses [29]. Physiologic predictors have included auditory evoked potentials [30], event-related potentials [31], and quantitative electroencephalograms [32]. Biochemical and endocrinology predictors include hypothalamic-pituitary-adrenal (HPA) axis measures [33], urinary 3-methoxy-4-hydroxyphenylglycol (MHPG) [34], and serotonergic measures in serum/platelets [35]. Imaging predictors have included pre-treatment patterns of regional glucose metabolism and blood flow measured with positron emission tomography (PET) [36], as well as structural and functional magnetic resonance imaging (fMRI) studies [37].

With rare exception, the previously noted studies have retrospectively examined patterns correlated to outcomes for a specific treatment. A few have tracked differences between responders and non-responders to two different treatments, for example drug *vs.* psychotherapy or drug *vs.* drug [38]. Numerous attempts at identifying genetic polymorphisms as predictors of treatment for MDD have been made [39]. So far, except for some polymorphisms directly influencing the pharmacokinetics of antidepressant drugs [40], no consistently replicated (and thus clinically relevant) candidates have emerged from candidate gene or genome-wide association studies [41]. This suggests that genetic information may need to be combined with other biomarkers and clinical variables for a more reliable prediction of response [42]. In addition, few previous studies have investigated genetic polymorphisms as differential predictors for different types of antidepressant treatments [43].

One of the strategic objectives of the National Institute of Mental Health (NIMH) is to develop better, more specific interventions for patients with mental illnesses; this approach is broadly known as ‘personalized medicine’. In the field of mental health, personalized medicine has come to encompass the moderators and mediators of treatment response, including biological, genetic, behavioral, experiential, clinical, and environmental factors [44]. Personalized medicine may gain additional importance with the increases in racial and ethnic diversity predicted by the US Census Bureau [45]. Before these NIMH goals and objectives were fully articulated, we developed the Emory Predictors of Response in Depression to Individual and Combined Treatments (PReDICT) study that commenced recruitment in January 2007.

### Aims

The primary aim of PReDICT is to identify predictors of remission with acute treatment for MDD; predictors comprise genetic, endocrine, immune, and personality measures, as well as baseline and early-treatment fMRI of the central nervous system. Predictors will be identified by utilizing sophisticated multivariate procedures to determine which variables have clinically important and statistically significant effects for the prediction of remission and response.

A secondary aim of the project is to identify predictors of recurrence of major depression during a 21-month follow-up after acute treatment. Predictors of remission will include baseline, treatment, and post-treatment measures, detailed below.

## Methods/design

### Overview

**Figure 1:**
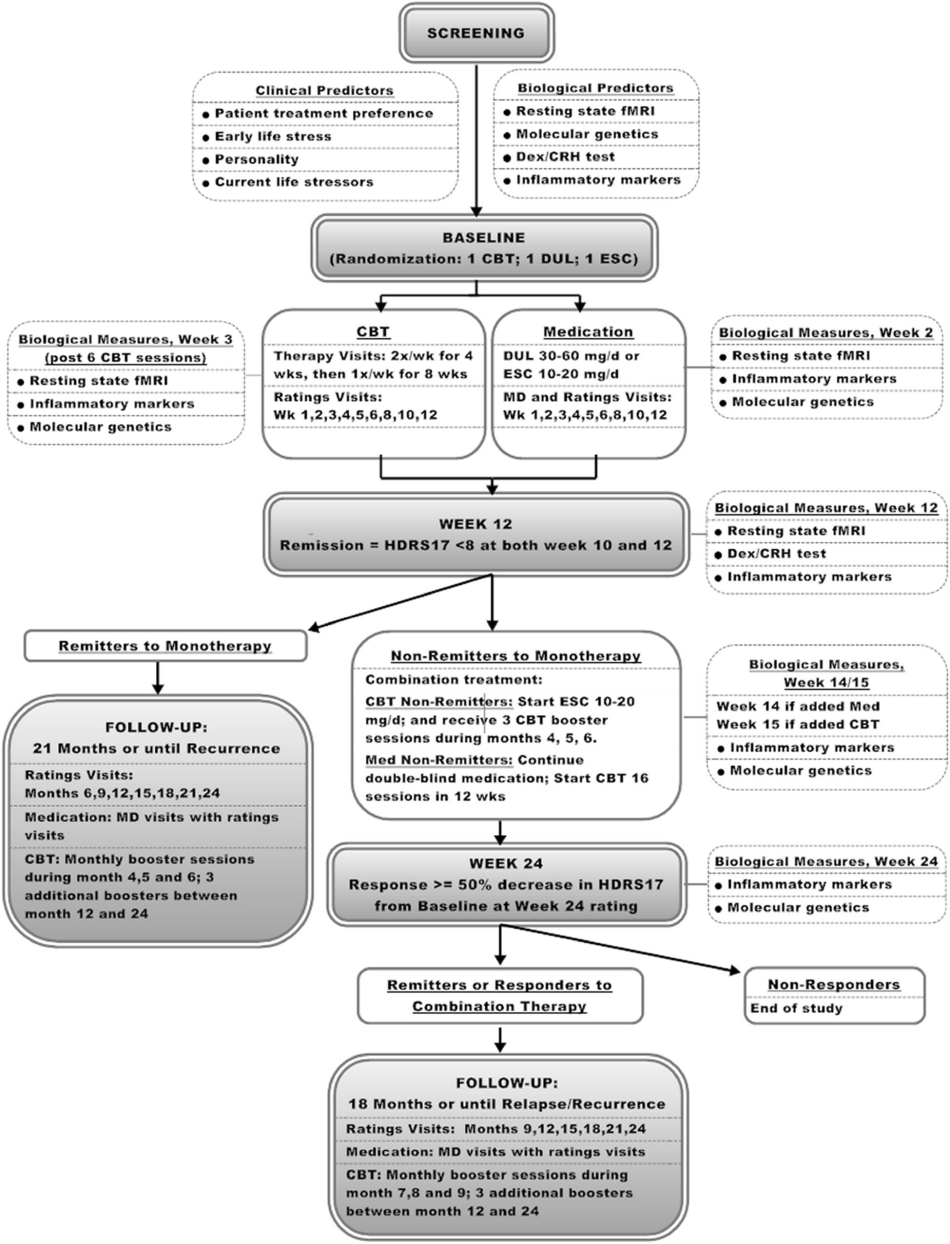
PReDICT study design.

Four hundred^a^ treatment-naïve patients with a primary diagnosis of MDD and HDRS-17 scores ≥18 at screening and ≥15 at the baseline visit are to be randomly assigned equally to one of three possible treatments: (1) a selective serotonin reuptake inhibitor (SSRI, escitalopram); (2) a serotonin norepinephrine reuptake inhibitor (SNRI, duloxetine); or (3) individual cognitive behavior therapy (CBT). Initial treatment consists of a 12-week course with one of these monotherapies. Patients who meet criteria for full remission of their MDD are followed for a 21-month follow-up phase to monitor for depression recurrence. Patients who do not remit after the 12-week monotherapy acute treatment phase are offered another 12 weeks of acute treatment, during which they receive combination therapy with study medication plus CBT. The overall study design is presented in Figure 1.

The study was designed and is being conducted in accord with the latest version of the Declaration of Helsinki [46]. The Emory Institutional Review Board and the Grady Hospital Research Oversight Committee gave ethical approval for the study design, procedures, and recruitment strategies (Emory IRB numbers 00024975 and 00004719).

### Study participants

The study’s total targeted enrollment is 400 18-65-year-old men and women who meet DSM-IV defined criteria for current major depressive disorder (MDD) and who have never previously received treatment for a mood disorder. Patients with acute, recurrent, or chronic MDD are included. The study excludes psychotic subtype of MDD, seasonal affective disorder, and pregnant or breast-feeding women. All interested patients undergo a telephone screening to assess preliminary eligibility, and potentially eligible patients are then scheduled for an in-office screening visit. Patients are not paid for participation during the acute phases, but they do receive the equivalent of approximately $5 per visit to offset travel-related costs. Patients entering into the follow-up phase receive $50 per visit to compensate for their time and inconvenience. The fundamental reason for examining treatment-naïve patients is to avoid confounding the biology of the illness with the effects of previous treatment, an effect that might result in persistent biological changes. Although the strictest definition of treatment-naïve would be patients who have not received a single dose of an antidepressant/psychotropic or a single session of formal individual psychotherapy, defining treatment naiveté so strictly is unnecessarily restrictive and reduces the feasibility of recruiting the targeted number of subjects. We have therefore operationalized previous treatment (and therefore study ineligibility) as treatment for MDD, dysthymia, or depressive disorder not otherwise specified with either: (1) a marketed antidepressant at a minimum effective dose for 4 or more consecutive weeks; or (2) four or more sessions of an established structured psychotherapy for depression, that is CBT, behavior therapy, interpersonal therapy (IPT), or behavioral marital therapy. Patients who have received supportive therapy or other forms of therapy are eligible, regardless of the duration of their previous psychotherapy course.

### Study sites

The study is being performed at three Atlanta sites associated with the Emory University School of Medicine Department of Psychiatry and Behavioral Sciences. The primary site, where all assessments are conducted in English, is located on the campus of Emory University, in suburban Atlanta. A second English-speaking satellite site, located in Stockbridge, GA, was added to the study in July 2011. This site is open 2 days per week, and operates with the same personnel and randomization and blocking schedule as the primary site. The third site is located in the International Medical Center (IMC) at Grady Memorial Hospital in downtown Atlanta. This site is staffed entirely by bilingual (English/Spanish) and bicultural personnel, who also participate in the study at the primary site. All assessments at the IMC site are conducted in Spanish, employing validated translations of the rating scales and self-reports. Instruments without a previously validated translation and CBT materials were translated and evaluated by an expert panel. A Spanish-language site was established to assure a more diverse and representative study population, recognizing that Hispanics are the largest and fastest growing minority group in the US [45]; this site employs a separate randomization and blocking schedule from the primary Emory site. The Dexamethasone-Corticotropin Releasing Hormone tests (Dex/CRH) (see below) are conducted in the Clinical Interaction Network (CIN) of the Atlanta Clinical and Translational Science Institute at both Emory and Grady Hospitals. Study fMRIs for patients treated through both sites are conducted at the Emory University Biomedical Imaging Technology Center.

### Screening and treatment assessments

**Figure 2:**
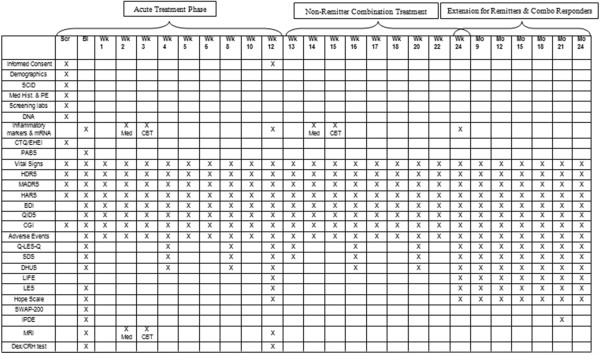
**Schedule of events for PReDICT study.** NOTE: Remitters to the acute (monotherapy) treatment phase enter into the extension follow-up phase beginning at week 12. BDI, Beck Depression Inventory; CGI, Clinical Global Impression; CTQ, Childhood Trauma Questionnaire: Dex/CRH, Dexamethasone Corticotropin Releasing Hormone test; DHUS, Daily Hassles and Uplifts Scale; DNA, Deoxyribonucleic acid; EHEI, Early Home Environment Interview; HARS, Hamilton Anxiety Rating Scale; HDRS, Hamilton Depression Rating Scale; IPDE, International Personality Disorders Examination; LES, Life Experiences Survey; LIFE, Longitudinal Interview Follow-up Evaluation; MADRS, Montgomery Depression Rating Scale; MRI, Magnetic resonance imaging; mRNA, Messenger ribonucleic acid; PABS, Patient Attitudes and Beliefs Scale; PE, Physical Exam; QIDS, Quick Inventory of Depressive Symptoms; Q-LES-Q, Quality of Life Enjoyment and Satisfaction Questionnaire; SCID, Structured Clinical Interview for DSM-IV; SDS, Sheehan Disability Scale; SWAP, Shedler-Westen Assessment Procedure, 2^nd^ Ed.

The schedule of assessments is presented in Figure 2. After signing the informed consent form, study participants meet with a staff member for an initial psychiatric interview. The results of this initial interview are then presented to a study psychiatrist, who subsequently conducts a 30 to 60-min diagnostic evaluation, including medical history and previous treatment history. Patients who remain eligible then complete the Structured Clinical Interview for DSM-IV (SCID) [47,48] administered by a trained clinical interviewer. Symptom severity is assessed by the SCID interviewer who also administers the HDRS-24 plus atypical items [49,50] using a structured interview guide [51], Montgomery-Asberg Depression Rating Scale (MADRS) [52,53], Hamilton Anxiety Rating Scale (HARS) [54], and Clinical Global Impression scale for Severity (CGI-S) [55]. Patients are then evaluated to ensure adequate physical health and to identify potential medical causes for a major depressive episode. This evaluation includes: a medical review of systems, physical exam, electrocardiogram and laboratory assessments (comprehensive metabolic panel, complete blood count, thyroid-stimulating hormone level, pregnancy test, and urinalysis and urine drug screen). Demographic variables and family history of psychiatric illness are collected via self-report. Childhood trauma history is assessed via the Childhood Trauma Questionnaire (CTQ) [56] and the Early Home Environment Interview (EHEI) [57]. Inclusion and exclusion criteria are listed below. Patients who meet all eligibility criteria undergo phlebotomy for measurement of inflammatory markers and extraction of mRNA and DNA. Subsequently, patients complete the fMRI and a half-day CIN outpatient hospital stay during which the Dex/CRH test is performed. The targeted time to complete all pre-treatment assessments from screening to randomization is 10 days. 

### Inclusion criteria

1. Male or female outpatients aged between 18 and 65 years old

2. Primary psychiatric diagnosis of DSM-IV-defined major depressive disorder

3. Total HDRS-17 score ≥18 at screening visit, AND ≥15 at randomization visit

4. Never previously treated for MDD or dysthymia, defined as:

a. Four or more consecutive weeks of an antidepressant at minimally effective dose, OR

b. Four or more sessions of an established structured psychotherapy for depression, i.e. CBT, BT, IPT, or behavioral marital therapy

5. Able to independently understand and provide written informed consent

6. Able to communicate fluently in either English or Spanish

### Exclusion criteria

1. Current DSM-IV defined psychotic disorder, eating disorder, dissociative disorder, obsessive compulsive disorder, or dementia

2. Any current primary DSM-IV disorder other than major depressive disorder

3. Lifetime history of DSM-IV defined bipolar disorder or schizophrenia

4. Current clinically important suicidal ideation requiring rapid initiation of treatment

5. Meeting DSM-IV criteria for alcohol or drug dependence within 12 months, or substance abuse within 3 months of randomization visit (excluding nicotine and caffeine)

6. Urine drug screen positive for drugs of abuse at screening visit

7. Any lifetime prior exposure to citalopram, escitalopram, or duloxetine

8. Any lifetime adequate medication treatment (≥4 weeks at minimal effective dose) for major depression or dysthymia

9. Any lifetime prior treatment with four or more sessions of an established structured psychotherapy for depression, i.e. CBT, BT, IPT, or behavioral marital therapy

10. Treatment with any dose (including less than minimally effective dose) of an antidepressant for any reason for ≥4 weeks for during the current episode

11. Use of any psychotropic medication (except hypnotics) within 1 week of the screening visit

12. Any use of fluoxetine within 8 weeks of the screening visit

13. Need for concurrent neuroleptic or mood stabilizer therapy

14. Currently pregnant or breast-feeding women

15. Any current acute or chronic medical disorder that would likely affect or preclude completion of the study

16. Clinically important neurological, inflammatory, autoimmune, endocrine, or other medical illness that could interfere with the conduct of the study or interfere with interpretation of study results, including clinically important abnormal screening laboratory results

17. Medical contraindications which would preclude treatment with escitalopram or duloxetine

18. Presence of any factors that would likely prevent the patient from completing 12 weeks of the study

19. Contraindications for MRI, such as pacemaker, aneurysm clips, or other implants

20. Unlikely to comply with the study protocol, as judged by a study psychiatrist

### Randomization

Following completion of the predictors assessments, patients participate in the baseline assessments and are randomized. Self-report measures of depression severity at this baseline visit include the Quick Inventory of Depressive Symptoms-Self Report (QIDS-SR) [58] and the Beck Depression Inventory (BDI) [59,60]. Quality of life is assessed with the Qualitative Life Enjoyment Satisfaction Questionnaire (Q-LES-Q) [61] and the Sheehan Disability Scale (SDS) [62,63]. Patients also complete an abbreviated version of the Patient Attitudes and Beliefs Scale (PABS) [64], to indicate their treatment preference and their beliefs about the causes of their depression, and the Life Experiences Survey [65] to assess important personal events over the previous 12 months. A blinded rater administers the HDRS-24, HARS, MADRS, CGI-S, and CGI-Improvement (CGI-I) [55]. To be randomized, patients must score ≥15 on the HDRS-17 total score at this visit.

A permuted block randomization pattern was generated prior to opening enrollment for the study. Randomized treatment assignments were individually printed and placed in sealed opaque envelopes by Emory employees uninvolved in the study. The randomization envelopes were stored in the research offices and opened sequentially at the time of each patient’s randomization visit. The treatment assignment was generated using randomized permuted blocks, stratified by site, to ensure equal allocation across treatment groups within the English- and Spanish-language sites and at all times throughout the study.

### Protocol treatments

#### Pharmacotherapy

A similar proportion of MDD patients respond acutely to any single class of antidepressant medications [66]. Meta-analyses using remission as the outcome criterion indicated a potential advantage for medications with dual effects on serotonin and norepinephrine (primarily venlafaxine) over SSRIs [67]. However, the number needed to treat to achieve an additional remission with an SNRI *vs.* SSRI is high, and of doubtful clinical importance. A pooled analysis of six phase II/III trials comparing duloxetine to an SSRI (fluoxetine or paroxetine) found no difference in remission rates between medication classes [68]. Meta-analyses of the efficacy of escitalopram have found it to be as good or better than SNRI treatment for MDD [69,70]. However, some patients clearly respond to certain antidepressants but not others, indicating the need to identify differential predictors of response for these treatments.

The patent-holding companies provide the medications at no cost to the study. Bulk-shipped medication is compounded in the Emory investigational drug service pharmacy and packaged in purple capsules. Each capsule contains either escitalopram oxalate (equivalent to 10 mg of escitalopram free base) or duloxetine HCl (equivalent to 30 mg of duloxetine free base). All patients are started on one capsule per day, dosed in the morning with food. If daytime sedation occurs, the psychiatrist may change dosing to bedtime. If the patient does not demonstrate clinically meaningful improvement by week 4, the dose is raised to two capsules per day, though the treating psychiatrist, based on the severity of the patient‘s symptoms, may raise the dose earlier if deemed necessary. If there is a plateau in response, or if remission is not achieved by week 6, the dose is increased to two capsules per day. If adverse events are sufficiently distressing to the patient, the dose can be lowered back to one capsule per day. Patients eligible for the follow-up phase of PReDICT are strongly encouraged to remain on medication through month 12, at which time the psychiatrist discusses with the patient the risks and benefits of discontinuing the medication given their respective number of prior untreated depressive episodes. Regardless of their decision to continue or discontinue medication at this stage, patients continue into the second year of follow-up. The physicians overseeing medication treatment are all board-certified psychiatrists or fourth-year psychiatry residents under close supervision of the lead study psychiatrist (BWD).

We will measure concentrations of escitalopram and duloxetine primarily to evaluate adherence but also to monitor individual subjects’ metabolism and clearance of the medications. The assay utilizes a validated liquid chromatography tandem mass spectroscopic method to assess concentrations of all commonly used antidepressants and has a limit of detection of 0.2 ng/mL [71]. Absolute recoveries vary from 88.9% to 119.6% and inter-assay imprecision varies from 3% to 13% at levels of 75 and 300 ng/mL, for all compounds. The method compares favorably with the HPLC-UV methods used previously in our laboratory [72]. Serum is collected at baseline, week 2 and week 12 (and week 14 and 24 if in combination treatment) to assess medication adherence. These samples will be batch processed after study completion; consequently, they will not be used to guide dosing decisions or identify non-adherent patients during their study treatment.

#### Cognitive behavior therapy (CBT)

CBT was selected as the non-pharmacological treatment for this study because of its well-documented clinical efficacy in depressed patients at all levels of depression severity and chronicity [12]. It offers specific advantages over other psychotherapies for a study of this type. Specifically, the therapeutic process has been validated and the general goals and procedures for each session are standardized with available published training manuals [73]. Training and competence measures can be evaluated to insure a consistent standard of care for all patients.

The CBT package employed in the PReDICT study utilizes a standardized treatment protocol [73]. CBT pursues symptomatic relief from depression through a systematic effort to change depressed patients’ automatic and maladaptive ways of thinking. At the heart of this approach is the assumption that distorted views about the self, the world, and the future maintain depressive affect. Patients first work at becoming aware of these thinking styles, and then learn ways of thinking differently so that their cognitive processes become more adaptive. These skills, when accompanied by affective arousal and practiced in the context of extra homework assignments, are an important engine of symptom resolution [74]. Patients are first asked to record or keep track of their thoughts, especially those accessed in the midst of problematic emotional situations. During the course of treatment, the therapist gradually moves the focus of therapy from these distorted cognitions about themselves, the world, and the future to identification and modification of the underlying depressogenic maladaptive cognitive processes or errors. Finally, the patient comes to understand the beliefs underlying the cognitive processes and self-statements, and the therapy focuses on helping the patient change the underlying erroneous beliefs.

The typical course of CBT comprises 12 to 20 1-h sessions; in this study, 16 sessions are provided. The sequence of therapy involves three stages. In the early stage (sessions 1 to 4) the emphasis is on establishing a therapeutic relationship with the patient, educating the patient about the cognitive model and emotional lability, setting goals, and identifying, eliciting, and evaluating automatic thoughts. The middle phase (sessions 5 to 12) involves a gradual shift towards the identification of dysfunctional beliefs and compensatory strategies the patient may be employing, helping the patient to: (1) identify dysfunctional cognitive processes (for example, overgeneralization, mind reading) and core beliefs or schema; and (2) practice skills at responding to and modifying depressogenic processes and schema. Tasks in the late stage of CBT (sessions 13 to 16) revolve around preparing the patient for termination, predicting high-risk situations relevant to relapse, and consolidation of learning self-therapy tasks. To complete the 16 visits during the 12 weeks of intervention, patients randomized to CBT see their therapist twice per week for the first 4 weeks and then weekly for the remaining 8 weeks. The number of sessions attended is tracked for each patient.

Doctoral-level and masters-level providers trained in the specific CBT protocol for the study will provide the therapy. Patients who complete at least 12 sessions of CBT will be considered to have completed the course of CBT. All CBT-treated patients continuing in the study after week 12 receive three booster sessions at monthly intervals over the first 3 months and another three booster sessions, each separated by at least 1 month, during the second year of follow-up. There is also one additional crisis session available to patients during each year of follow-up. All therapy sessions are videotaped, and an independent off-site CBT expert at Beck’s Institute of Cognitive Therapy rates selected sessions of all therapists’ competence in Beck’s type of CBT [73].

#### Acute monotherapy phase assessment visits

All patients are seen for symptom severity ratings visits weekly for the first 6 weeks and bi-weekly for the remaining 6 weeks of the acute phase. At each visit, the patient completes the BDI and QIDS, and undergoes assessment with the HDRS, MADRS, and HARS with a blinded rater. All patients also meet with a study psychiatrist for assessment of CGIs, concomitant medications, adverse events, and safety. Thus, all patients, regardless of treatment assignment, meet with a study psychiatrist at all ratings visits throughout the study. Ratings visits are scheduled to coincide with a treatment visit (whether CBT or medication) to minimize patient time demands.

For patients randomized to medication, the study psychiatrist also assesses medication compliance, response to treatment, and need for dose adjustment at each of the ratings visits. For all patients, the psychiatrist limits the visit to a maximum of 20 min, unless an urgent clinical situation requires greater intervention. The total time the psychiatrist spends with the patient at each visit is recorded, and psychiatrists are instructed to adhere to the *Clinical Management Manual* developed by Fawcett *et al*. [75].

Patients who do not remit (that is, those with an HDRS-17 total score of ≥8 at either week 10 or week 12) following 12 weeks of monotherapy are offered the option to enter an additional 12-week course of combination treatment. Patients who initially received a medication as monotherapy continue on that medication in a double-blind manner, and begin the 16-session course of CBT. Patients who initially received CBT will have open-label escitalopram added to their treatment, following the same dosing guidance as described for the monotherapy treatment. We chose to use open-label medication in this phase due to inadequate power to identify differences between randomized treatments. These patients also meet with their CBT therapist once per month for a total of three monthly booster sessions over this 12-week period. The visit structure for this combination phase treatment replicates that of the monotherapy phase: weekly ratings visits for 6 weeks, followed by bi-weekly ratings visits for the next 6 weeks. At the end of this 12-week combination treatment period (that is, week 24 from baseline), patients are assessed for response to the combination treatment (see below). Those who remit or respond (defined below) can continue into the long-term follow-up phase (described below) for an additional 18 months, following the same visit schedule as the monotherapy phase remitters.

#### Long-term follow-up visits

Patients who are remitters at the end of the acute phase, or who are remitters or responders at the end of combination treatment are eligible to enter into long-term follow-up. These patients return for an assessment at the end of each 3-month period following the end of their acute treatment (that is, week 12 for monotherapy remitters, week 24 for remitters/responders to combination treatment). The assessments at these follow-up visits consist of a Longitudinal Interval Follow-up Evaluation (LIFE) interview [76], a clinical interview by a psychiatrist, clinical ratings on the HDRS, MADRS, and HARS by a trained blinded rater, and self-report measures. Patients continue in the follow-up phase until: (1) 2 years from study baseline; or (2) depressive recurrence (defined below). If, between scheduled follow-up phase visits, the patient reports clinically important return of symptoms and functional impairment such that they desire additional or alternative antidepressant treatment, they are considered to be at risk for recurrence, and a LIFE will be conducted as soon as possible.

#### Major endpoints

##### Remission

Remission, the study’s primary outcome measure, is defined as a HDRS-17 item total score <8 at both the week 10 and week 12 ratings visits for patients in the acute monotherapy phase. For patients who enter the combination treatment phase, remission is defined as a HDRS-17 item total score <8 at both the week 22 and week 24 ratings visits. Remitters from either the monotherapy or combination treatment phases are eligible to enter the long-term follow-up phase (21 months for monotherapy remitters; 18 months for combination remitters), returning every 3 months for assessments or until depression recurrence.

##### Response

Response is defined as ≥50% reduction from the baseline HDRS-17 item total score, and response will be used as a secondary outcome measure. Response is determined based on the HDRS-17 item rating at the week 12 and week 24 visits. For the non-remitters at week 12, the week 24 response/non-response status is used to determine further study participation. Responders at week 24 (that is, those patients who did not remit during the initial 12-week monotherapy phase, but who meet criteria for response at the end of the combination treatment phase) are offered the option to enter into the long-term follow-up phase. These patients return every 3 months for assessments for 18 months, or until depression recurrence, following the same follow-up schedule as the remitting patients. Recurrence is defined as the occurrence of any one of the following four criteria during the long-term follow-up phase: (1) Meeting full criteria for a major depressive episode: determined through a LIFE score of 3 for major depression, administered by a blind rater; (2) A 17-item HDRS ≥14 score in 2 consecutive weeks: blinded raters will administer the HDRS at the scheduled follow-up visits. A patient scoring ≥14 at a follow-up visit will be asked to return the subsequent week for a repeat rating. If that rating is also ≥14, the patient is considered to have had a recurrence; (3) A 17-item HDRS score ≥14 at any follow-up visit, and at which the patient requests an immediate change in treatment for their depressive symptoms; and (4) High current risk for suicidality (as assessed by the study psychiatrist) that warrants urgent intervention.

#### Predictors

##### Functional magnetic resonance imaging

Recent developments in image acquisition and data analysis now suggest that resting state studies can be reliably acquired using blood oxygen level-dependent (BOLD) fMRI [77-79]. Temporally correlated fluctuations in regional activity can be defined using both model-driven (correlations with specific seed regions) and data-driven approaches (that is, independent components analysis or self-organizing maps). These fluctuations agree with the concept of functional connectivity, a descriptive measure of spatio-temporal correlations between spatially distinct regions. Using such methods, decreased low-frequency correlations have been reported in depression [80-82]. These first results suggest fMRI can identify network abnormalities comparable to those previously identified using PET [36,83]. Critical to the goal of personalized treatment, resting BOLD fMRI methods can be implemented in scans of individual patients. Resting-state BOLD fMRI scans are acquired prior to initiating treatment and at a fixed, early time-point specific for each treatment. A third fMRI is performed at the completion of the 12 weeks of acute phase treatment, to serve as a predictor of recurrence during maintenance treatment. Pre-treatment scan patterns derived using multivariate analyses and associated with the treatment outcomes will be used to determine whether pretreatment brain patterns can distinguish among outcome groups [82,84]. A second fMRI scan, acquired early in the treatment course, will be used to assess the likelihood of response to the specific treatment assigned. These analyses will serve as a first step towards defining brain-based subtypes predictive of differential treatment outcome in major depression. The data from these studies will also be entered into more complex algorithms integrating imaging findings with behavioral, environmental, biochemical, and genetic information for individual patients.

#### Neuroimaging methods

##### Imaging acquisition

All images are acquired using a 3-Tesla whole-body MR system (Siemens MAGNETOM TIM Trio, Siemens Medical Solutions, Erlangen, Germany). The MR imaging protocol includes: (1) a 3-D anatomic volume (8 min); (2) resting-state functional connectivity (7.5 min); and (3) diffusion tensor imaging (DTI) (30 min); acquisition for a total of under 60 min of scanning. The anatomic imaging will be conducted for anatomic reference in fMRI data analysis and volumetric analysis [85]. The DTI images will be used to refine selection of regions-of-interest (ROI) for model-based analyses [82,86,87]. The baseline/resting fMRI acquisition will be used to measure region connectivity. The Positive and Negative Affective Scale will be administered before and after the scanning session to assess emotional states at the time of the fMRI [88].

##### Molecular genetic predictors

We collect whole blood for DNA extraction, and for DNA and lymphoblast banking at the Rutgers Cell & DNA Repository ( http://www.rucdr.org/) from every consenting patient at the screening visit. DNA will be used for genotyping genetic polymorphisms, mostly single nucleotide polymorphisms (SNPs), in a series of candidate genes that have emerged from previous human or animal studies or the ongoing genome-wide association studies thought to be relevant for antidepressant treatment response, including genes from the HPA-axis, monoaminergic systems, or neurotrophic systems [89]. The study was not powered to test for genome-wide association; however, data from it might be useful in meta-analyses across studies. The main intent of the DNA analyses is to examine hypothesized molecular genetic predictors in conjunction with other clinical and biological predictors to identify subtypes of patients more likely to respond to or remit with one of the three study treatments. DNA is also collected for future investigation of epigenetic modifications, including DNA methylation [90]. We extract RNA from whole blood collected in Tempus blood RNA tubes (Applied Biosystems) at the baseline visit after randomization and after 2 and 12 weeks of treatment. Analyses measure the expression of specific candidate transcripts encoding chaperone and co-chaperone proteins of the glucocorticoid receptor, using real-time PCR as well as gene-expression microarrays for a more comprehensive characterization of blood gene expression profiles [91]. We also assess the correlation of these gene-expression patterns with other putative biomarkers (for example, the Dex/CRH test and inflammatory markers) and test whether they can be used to predict or monitor treatment response [92].

##### Neuroendocrine function

The Dex/CRH test has been used to assess HPA axis function in depressed patients [93]. This test involves oral administration of dexamethasone at 2300 h the night prior to the assessment, followed by intravenous CRH administration at 1500 h the following afternoon. The CRH can be administered as a fixed dose of 100 mg of human CRH or 1 mg/kg of ovine CRH. At 1500 h, prior to the CRH infusion, and at set intervals for the following 2 h, plasma samples are collected for cortisol and adrenocorticotropic hormone (ACTH). In healthy subjects, there is little increase in concentrations of cortisol or ACTH after administration of CRH because the CRH can only minimally override the HPA-suppressing effects of dexamethasone. In many patients with MDD, however, this test induces substantial increases in plasma ACTH and cortisol concentrations [94]. The mechanism behind this effect is presumed to be impaired signaling of the glucocorticoid receptor (GR) [95]. Reduced GR sensitivity diminishes the ability of dexamethasone to suppress the HPA axis at the level of the pituitary, and it also reduces the inhibition of CRH and arginine vasopressin release from the paraventricular nuclei of the hypothalamus, consequently enhancing the stimulatory effects of the exogenously administered CRH [96].

Use of the Dex/CRH test in several studies has demonstrated HPA axis dysregulation during a major depressive episode, and normalization of the axis after recovery [95,97,98]. Sustained non-suppression of the HPA axis in MDD patients undergoing the Dex/CRH test predicts a worse prognosis for treatment response [94,99]. Some data also suggest that this test may predict the risk of depressive relapse [100]. The Dex/CRH test is well tolerated in medically healthy subjects [101].

For the PReDICT protocol, we conduct the Dex/CRH test immediately prior to randomization and at the end of the 12-week acute phase treatment period, using the protocol described by Heim *et al*. [102]. We used human CRH for study participants during the first 2 years of the study. However, the manufacturer subsequently experienced production difficulties, causing us to switch to ovine CRH for the remainder of the study.

##### Inflammatory markers

A plethora of studies have confirmed that medically healthy subjects with major depression demonstrate increased circulating concentrations of the proinflammatory cytokines tumor necrosis factor (TNF)-alpha, interleukin (IL)-1-beta, and IL-6 as well as acute phase reactants, especially C-reactive protein (CRP) [103-105]. Associations between depression and increased proinflammatory cytokines or CRP have been apparent across the adult life span, whether comparisons are made between clinically depressed patients and matched controls [106] or whether they arise from large population-based studies [107,108]. Recent investigations have reported positive correlations between levels of various inflammatory mediators and depressive symptom severity; furthermore, the association between immune activation and depression appears to be robust enough to be detectable in the context of mild depressive symptoms that do not meet criteria for major depression [109]. Supportive of the idea that inflammation is involved in the broader pathophysiology of MDD, therapeutic administration of cytokines promotes the development of frank depressive symptoms and syndromes. For example, 40% to 50% of patients receiving chronic treatment with interferon-alpha (which induces the production/release of IL-6, IL-1b, and TNF-alpha) develop clinically important depressive symptoms or meet criteria for major depression within 3 to 6 months of treatment initiation [110]. Relevant to the pathophysiology of major depression, proinflammatory cytokines are potent activators of corticotropin-releasing hormone (CRH) pathways (that is, the HPA axis and sympathetic nervous system) [111]. Cytokines appear capable of inhibiting glucocorticoid receptor functioning [112] and of diminishing CNS serotonin signaling capacity by reducing tryptophan bioavailability and by increasing presynaptic serotonin reuptake activity [113,114].

IL-6 and CRP assessment: To further clarify whether IL-6 and CRP are predictors of treatment response (early or final), plasma collected at baseline, at a point early in treatment, and at week 12 will be analyzed for concentrations of IL-6 and CRP. Plasma concentrations of IL-6 will be determined in duplicate using sandwich ELISA according to manufacturer‘s protocol (R & D Systems, Minneapolis, MN, USA) [115]. The mean inter- and intra-assay coefficients of variation for control samples in this assay are reliably 10% or less [116].

Plasma CRP will be assessed with a high-sensitivity turbidimetric assay based within the Emory University Hospital Clinical Laboratories. Sensitivity of the assay is rated at 0.18 mg/L, detection limits of the assay are 0.2 to 80 mg/L, and functional sensitivity (at 20% CV) is 0.2 mg/L.

##### Personality

Prior research suggests that personality variables, including personality disorders (PDs), predict the longitudinal course of MDD in treatment-seeking patients [117,118], although data on personality predictors of response to specific treatments are sparse [119]. Perhaps the central question at the heart of current research on the classification of personality pathology, which bears on predictors of treatment outcome, is how best to conceptualize personality: as categorical PDs; as sums of numbers of PDs or PD criteria met [117]; as dimensional representations of syndromes (for example, personality prototypes, for which patients can vary in the degree to which they match a given diagnosis [120]); or as dimensional traits (for example, neuroticism, emotional dysregulation, or emotional avoidance) [121,122].

We addressed these issues in this study by using measures that cover the landscape of personality and PD diagnosis: (1) The Shedler-Westen Assessment Procedure, 2^nd^ edition (SWAP-II), an instrument developed for use by clinically expert observers (much like the HRSD for depression), which yields dimensional DSM-IV PD diagnoses, empirically-derived PD syndromes, empirically-derived traits, and empirically-derived scales that describe patients whose MDD did and did not respond to various forms of treatment in naturalistic settings [123]; and (2) the International Personality Disorders Examination (IPDE) [124], a semi-structured interview that can yield categorical and dimensional data on both the DSM-IV and ICD-10 PDs.

##### Clinical and demographic variables

Clinical and demographic variables are collected by self-report measures and structured interviews. Demographic data on gender, age, race, ethnicity, education level, marital status, employment status, and living situation are collected from an intake form completed at the screening visit. Family psychiatric history is collected using a self-report form listing major Axis I diagnoses and suicide, and inquiring which, if any, family members were affected.

Among the data for clinical variables collected are the self-reported history of childhood trauma using the CTQ and EHEI, and recent important life events from the Life Experiences Survey [76]. The self-report Goals scale [125] and Combined Hassles and Uplifts scale [126] assess current levels of hopelessness and frustration and are collected at baseline and at intervals throughout the acute and follow-up phases. Depression and anxiety severity are assessed by both clinician interview (HDRS, MADRS, HARS) and self-report forms (QIDS, BDI). History of substance abuse and dependence and presence of co-morbid psychiatric conditions are obtained from the SCID interview.

#### Data management

A local area network resident relational database (Microsoft Office Access 2010, Microsoft, Redmond, WA, USA) serves as the secure repository for study data. The relational structure of the database has been optimized via conformity to established guidelines for data normalization [127]. All tables in the relational database fulfill criteria for third normal form at a minimum, thereby ensuring structural optimization of the database [127].

The central tables in the relational database include: (1) [Subject] table with one row per participant and each row uniquely identified by a HIPAA-compliant subject number [128]; and (2) [Subject Contact] table with one row per participant visit and each row uniquely identified by the combination of the subject number and contact date. Other dependent tables store data regarding phase of study participation and collection and tracking of biological samples including aliquot management and recording of results. Database security is ensured by hierarchical password-secured access beginning with password-restricted access to the network followed by password-restricted access to the database itself. The database security helps to maintain in the masking of research staff to randomized treatment by restricting access to certain segments of the data.

The relational database also aids in assuring data integrity. Real-time (point-of-entry) data validation is implemented by utilizing: (1) data rules imposed by the table-to-table relationships of the normalized data (for example, it is impossible to enter rating scale data on an erroneous visit date); (2) item-level range checks and compatibility checks; (3) double entry of all scales with automated checking for discrepancies; and (4) summary checks of psychometric scales utilizing mirrored (redundant) data entry. Other data validation measures include automated import of laboratory results (eliminating transcription errors) and regular review of automatically-generated data compatibility reports (for example, report flags as a possible error when CGI and HDRS values for a patient visit appear to be incompatible, triggering review of previously entered data).

Finally, the optimized structure of the relational database has enabled the *a priori* development and implementation of queries and reports that service routine outside reporting needs (for example, reporting to study sponsor, data safety monitoring board, institutional review board), internal tracking of progress toward completing study objectives, and export of HIPAA-compliant study data for import into external statistical software packages for data analysis. In addition, the normalized structure of the database simplifies fulfillment of *ad hoc* data requests for additional novel purposes including preliminary data for other applications, abstract submission, and data sharing.

#### Data analyses

The primary data analyses consist of two phases: (1) identification of significant predictors of treatment response, both overall and treatment-specific; and (2) building of a comprehensive model of treatment response similar to those which predict remission and response to treatment as well as those who suffer a recurrence [129]. The first phase will use various variable selection methods (for example, recursive partitioning [130,131]), as well as different measures of discriminating ability (for example, C statistic or ROC [132], net reclassification improvement [133]), to determine the best set of predictors that are both statistically significant and clinically important using predefined importance criteria. The building of the prognostic model will use established techniques [134] to create a model of treatment response that can then be tested in future research, including quantifying predictors, calibrating the model, and possibly adapting it to the clinic using a points method. All analyses will be performed in Stata 12.

#### Statistical power/sample size

There are no established ways to determine sample size for the identification of possible predictors of an outcome, as it is a fact-finding endeavor and does not test specific *a priori* hypotheses. However, the issue of clinical importance is much more paramount in a study of this nature (that is, while statistically significant predictors are of interest, it is also necessary to determine if the predictors of interest result in a large enough absolute or relative difference in response between those with and without the predictor to be clinically meaningful) [135]. In addition, it is clear that smaller but statistically significant effects will likely be difficult to replicate and thus reduce the validity of the model for future use. Given the sample size we intend to recruit over the time period specified, we will illustrate the effect sizes for treatment remission versus non-remission, as very few data on differential treatment response are available. For these calculations, we assume a 40% remission rate. The detectable difference is defined as the difference (either mean or proportion) between the population and the treatment remitters detectable with the given sample size, a Type I error rate (α) = 0.01, and a statistical power of 0.80. We chose an α = 0.01 because the nature of the model building will require the testing of multiple predictors. Population values are determined from various published and unpublished data (Table 1). 

**Table 1:** Sample effect size calculations for selected variables

		**Monotherapy treatment remission (*n*** **= 400)**		**Combination treatment remission (*n*** **= 240)**	
**Variable of interest**	**Observed value in the population [mean (SD) or % positive]**	Detectable difference*	**Effect size (difference relative to variation)**	Detectable difference*	**Effect size (difference relative to variation)**
Endpoint HDRS score	16.0 (4.9)	−1.9	0.39	−2.5	0.51
Serotonin transporter gene carriers (minor allele carriers/Caucasian)	59.9%	17%	0.31	22%	0.40
Elevated C-reactive protein (>0.22 mg/dl)	21%	13%	0.36	16%	0.48
fMRI brain score	44.1 (4.3)	1.7	0.40	2.2	0.51
Baseline Dex/CRH response (cortisol AUC, (ng*min)/mL)	2730.6 (266)	104.6	0.39	136.1	0.51
Interleukin-6 (pg/mL)	6.3 (1.4)	0.6	0.43	0.7	0.50

Thus a sample size of 400 will be adequate to detect moderate to large effect sizes in treatment remission predictors [136]. Although the sample sizes for differential remission by treatments will be less, with a projected overall remission rate of 30% in any group, there will be approximately 40 remitters in each group to detect the statistically significant interactions. These are reasonable numbers for the detection of large interaction effect sizes [136].

#### Data safety monitoring board (DSMB)

Given that all treatments provided in the study are standard first-line treatments for MDD with well-established safety and tolerability profiles, the DSMB (a four-member panel of experienced psychiatry researchers) conducts reviews at yearly intervals during the study. Reports to the DSMB include all adverse events and any potential confidentiality breaches occurring during the study. Additionally, all serious adverse events that occur in the study are reported to the DSMB within 1 week of their occurrence. Because this is not a comparative efficacy trial, but rather a study of the moderators and mediators of standard first-line treatments at the level of the individual, the DSMB does not routinely receive efficacy outcome data, though such data are provided if requested.

## Discussion

Previous treatment with psychotherapy or medication may impact subsequent response to treatment and may produce persisting biological, behavioral, cognitive, and emotional changes [137]. Thus, we chose to examine predictors of treatment outcomes in patients never previously treated for depression. To make the trial reflect real-world decisions, we examined not only medication versus psychotherapy, but two different medications; all three treatments are currently considered potential first-line interventions. The design considered the primary treatment decisions to be made from the start of care including: (1) whether to initiate treatment with medication or psychotherapy; and (2) if a medication option is selected, which is the most appropriate specific class of medication. Thus, we designed a three-armed trial to evaluate clinical, biological, genetic, and personality factors that may predict outcomes to common first-line treatment options for MDD. The study was further designed to expect that while individual measures might prove predictive, the more likely outcome would be that a combination of clinical, imaging, and genetic markers would predict outcomes to individual treatments. The study design and unique patient population we have chosen has limitations, and alternative designs were carefully considered. We did not include a placebo control group or extended placebo lead-in because it would undoubtedly result in a marked reduction in the number patients willing to enter the study. To maximize generalizability and feasibility, we chose our medication treatments from the classes of antidepressants most commonly used to treat depression; those classes are SSRIs, SNRIs, and bupropion. Because anxiety disorders are highly co-morbid with major depression, and because the presence of a co-morbid anxiety disorder (other than OCD) was not an exclusion criterion for entry into the study, bupropion was not selected. Unlike SSRIs and SNRIs, bupropion does not have an FDA-indication for the treatment of anxiety disorders, and is associated with poorer response than SSRIs in treating depressed patients with high levels of anxiety [138].

Among the SSRIs, we opted for escitalopram because it is the most selective SSRI, having no or very little effects on norepinephrine or dopamine reuptake. In contrast, paroxetine is an antagonist of both the serotonin transporter (SERT) and norepinephrine transporter (NET) [139]. Escitalopram has the lowest likelihood of drug-drug interactions of any of the SSRIs [140]. Adverse effect rates with escitalopram are lower than with citalopram, which we expected to benefit patient retention in the study. Although one of the antidepressants clearly needed to be an SSRI, arguments could be made to replace duloxetine with any of the following: venlafaxine, mirtazapine, bupropion, nefazadone, a tricyclic antidepressant (TCA), or a monoamine oxidase inhibitor (MAOI). We opted against mirtazapine, nefazadone, TCAs, and MAOIs due to issues of lower tolerability and frequency of clinical use. Duloxetine was selected over venlafaxine as the SNRI for several reasons. First, the FDA-approved dose range of venlafaxine is much broader, and therefore less comparable to escitalopram. Second, venlafaxine has a more adverse cardiovascular toxicity profile, and it may have a greater overdose liability than duloxetine [141]. Finally, at low doses venlafaxine acts primarily through SERT inhibition, with higher doses required to achieve NET inhibition [142,143].

Interpersonal psychotherapy is another form of psychotherapy we could have included, but we did not possess the statistical power to add another treatment arm, and the supporting data for the efficacy of CBT are more extensive. Other limitations include the omission of geriatric depression - necessary because of the growing evidence suggesting that vascular depression, which accounts for a sizeable percentage of the geriatric depressed population, is truly a distinct neurobiological entity [144]. Also excluded are patients with clinically important co-morbid medical disorders or substance abuse, children and adolescents, and those with psychotic depression. Although these patients are of interest, we believe that the benefits of keeping our patient characteristics as free of potential confounding variables as possible will maximize our ability to examine our primary objectives.

We believe this study design best allows for assessment of the effects of several potentially important moderators and mediators of treatment outcomes, as well as the interactions between moderators, in a sample unconfounded by previous treatment effects. The results of this study should inform clinical treatment decisions and identify future research approaches to further improve the care of depressed patients.

## Trial status

Recruitment for this trial was ongoing at the time this manuscript was submitted and is expected to continue through March 2013.

## Endnotes

^a^We originally intended to recruit 600 patients for PReDICT, but subject recruitment efforts with the current inclusion/exclusion criteria resulted in the reduction to 400 as the expected total number of subjects that will be successfully recruited and randomized to participation in this project.

## Abbreviations

ACTH: Adrenocorticotropic hormone; AFSP: American Foundation for Suicide Prevention; AUC: Area under the curve; BDI: Beck Depression Inventory; BOLD: Blood oxygen level-dependent; BT: Behavioral therapy; CBT: Cognitive behavioral therapy; CGI-I: Clinical Global Impression of Improvement; CGI-S: Clinical Global Impression of Severity; CIDAR: Center for Intervention Development and Applied Research; CIN: Clinical interaction network; CNS: Central nervous system; CRH: Corticotropin-releasing hormone; CRP: C-Reactive protein; CTQ: Childhood Trauma Questionnaire; CV: Coefficient of variation; Dex/CRH: Dexamethasone/corticotropin-releasing hormone; DNA: Deoxyribonucleic acid; DSMB: Data safety monitoring board; DSM-IV: Diagnostic and Statistical Manual, 4^th^ edition; DTI: Diffusion tensor imaging; DUL: Duloxetine; EHEI: Early Home Environment Interview; ELISA: Enzyme-linked immunosorbent assay; ESC: Escitalopram; FDA: Food and Drug Administration; fMRI: Functional magnetic resonance imaging; GCRC: General clinical research center; GSK: GlaxoSmithKline; HPA: Hypothalamic pituitary adrenal; HPLC: High performance liquid chromatography; HARS: Hamilton Anxiety Rating Scale; HDRS: Hamilton Depression Rating Scale; HIPAA: Health Insurance Portability and Accountability Act; HPLC-UV: High-pressure liquid chromatography with UV detector; ICD-10: International Statistical Classification of Diseases and Related Health Problems, 10th revision; IMC: International Medical Center; IPDE: International Personality Disorders Examination; IPT: Interpersonal therapy; IL: Interleukin; LIFE: Longitudinal Interview Follow-up Evaluation; MADRS: Montgomery-Asberg Depression Rating Scale; MAOI: Monoamine oxidase inhibitor; MD: Doctor of medicine; MDD: Major depressive disorder; Med: Medication; MHPG: 3-methoxy-4-hydroxyphenylglycol; MRI: Magnetic resonance imaging; NARSAD: National Alliance for the Research of Schizophrenia and Depression; NET: Norepinephrine transporter; NIDA: National Institute on Drug Abuse; NIDDK: National Institute of Diabetes and Digestive and Kidney Diseases; NIH: National Institutes of Health; NIMH: National Institute of Mental Health; OCD: Obsessive compulsive disorder; PABS: Patient Attitudes and Beliefs Survey; PCR: Polymerase chain reaction; PD: Personality disorder; PET: Positron emission tomography; PHS: Public health service; PReDICT: Prediction of response in depression to individual and combined treatments; QIDS-SR: Quick Inventory of Depressive Symptoms - self report; Q-LES-Q: Quality ofLife Enjoyment and Satisfaction Questionnaire; RNA: Ribonucleic acid; ROC: Receiver-operating characteristics; ROI: Region of interest; SDS: Sheehan Disability Scale; SERT: Serotonin transporter; SCID: Structured Clinical Interview for DSM-IV; SNP: Single nucleotide polymorphism; SNRI: Serotonin norepinephrine reuptake inhibitor; SR: Self-report; SSRI: Selective serotonin reuptake inhibitor; SWAP: Shedler-Westen Assessment Procedure; TCA: Tricyclic antidepressant; TNF: Tumor necrosis factor; US: United States.

## Competing interests

In the past 5 years, the authors report the following: BWD has received honoraria for consulting work with Bristol-Myers Squibb, Imedex LLC, Medavante, and Pfizer. He has also received research support from Astra Zeneca, Bristol-Myers Squibb, Evotec, Forest, GlaxoSmithKline, NIMH, Novartis, Ono Pharmaceuticals, Pfizer, Takeda, and Transcept. EBB has received grant support from Pharma-Neuroboost and is co-inventors on the following patent applications: Means and methods for diagnosing predisposition for treatment emergent suicidal ideation (TESI). European application number: 08016477.5 International application number: PCT/EP2009/061575; FKBP5: a novel target for antidepressant therapy. International publication number: WO 2005/054500 and Polymorphisms in ABCB1 associated with a lack of clinical response to medicaments. International application number: PCT/EP2005/005194. JFC has received research support from NIDA, NIMH, NARSAD, Roche, and Seaside Therapeutics. MK has consulting agreements with Eagle Pharmaceutical and St Jude Medical Inc., and receives book royalty income from McGraw-Hill/Irwin. He also serves on the Clinical Advisory Board for the Shriners Hospital. Dr Kutner serves on an NIDDK DSMB for a multicenter trial of calcitriol compared to high and low dose cholecalciferol for the treatment of vitamin D insufficiency in patients with advanced chronic kidney failure. CBN has received research support from the NIH and Agency for Healthcare Research and Quality. He has served as a consultant to Xhale, Takeda and SK Pharma. He has been a stockholder in CeNeRx Biopharma, NovaDel Pharma Inc., PharmaNeuroboost, Revaax Pharma, and Xhale. He has had additional financial interests in Corcept, CeNeRx BioPharma. PharmaNeuroboost, Novadel Pharma, and Revaax. He has served on the scientific advisory boards of American Foundation for Suicide Prevention (AFSP); AstraZeneca, CeNeRx Biopharma, Forest Labs, Janssen/Ortho-McNeil, Mt. Cook Pharma Inc., NARSAD, NovaDel Pharma, Inc., Pharma-Neuroboost, Quintiles, and the Anxiety Disorders Association of America. He has served on the Board of Directors for the AFSP, George West Mental Health Foundation, NovaDel Pharma, Inc., and Mt. Cook Pharma Inc. Dr Nemeroff holds a patent on the method and devices for transdermal delivery of lithium (US 6,375,990 B1) and the method to estimate drug therapy via transport inhibition of monoamine neurotransmitters by *ex vivo* assay (US 7,148,027B2). JDN has received lifetime research support from Eli Lilly, Glaxo SmithKline (GSK), Janssen, the National Alliance for Research on Schizophrenia and Depression (NARSAD), the National Institutes of Health (NIH), and Wyeth. He has served on speakers’ bureaus or received honoraria from Astra-Zeneca, Eli Lilly, GSK, Pfizer, and Wyeth. He has served on advisory boards for GSK. MJO reports research support from NIH, Lundbeck A/S, Cyberonics, Eli Lilly, Ortho- McNeil Janssen, AstraZeneca, Dainippon Sumitomo Pharma, SK Life Sciences, and Sunovion Pharmaceuticals. He has served as a consultant to H Lundbeck A/S, and RJ Reynolds, and has a patent for a method of assessing antidepressant drug therapy via transport inhibition of monoamine neurotransmitters (US 7,148,027 B2). TWWP has received research funding from NIH and NARSAD, and has also received funding from GSK to examine gene expression patterns in patients with major depression. He also serves on a scientific advisory board for Questcor Pharmaceuticals. JCR has received research support from Abaxis Inc., Abbott, Beckman Coulter, NIH, Roche Diagnostics, and Waters, Inc. DW is a developer and copyright holder of the Shedler-Westen Assessment Procedure (SWAP-II), an instrument used in this study for personality assessment and diagnosis. The SWAP-II is likely to have commercial applications, available at http://www.swapassessment.org, although no funding was provided by any commercial entity for this research. WEC is an officer of Hugarheill enf, an Icelandic company dedicated to prevention of depression, and he receives book royalties from John Wiley and Sons. He is a consultant to the George West Mental Health Foundation that oversees Skyland Trail, a residential treatment facility in Atlanta, GA. HSM holds intellectual property in the field of deep brain stimulation for depression and is a consultant for St Jude Medical, Inc. MMG, BK, MEK, and VAR report no competing interests.

## Authors’ contributions

BWD led the manuscript development and serves as the lead study psychiatrist. EBB, JFC, MMG, MEK, BK, MK, JDN, MJO, JCR, and DW all contributed to the study design and wrote sections of the manuscript related to their area of expertise. VAR is the lead psychiatrist for the IMC Spanish-speaking clinic site and contributed to the sections of the manuscript related to that site. CBN conceived of the project, led the study development process and served as the project‘s initial principal investigator. WEC worked with BWD in the manuscript development, and he and HSM wrote the sections of the manuscript related to their area of expertise and serve as the principal investigators for the PReDICT study. All authors edited the manuscript and approved the final version.

## References

[B1] Kessler RC Berglund P Demler O Jin R Merikangas KR Walters EE Lifetime prevalence and age-of-onset distributions of DSM-IV disorders in the National Comorbidity Survey Replication Arch Gen Psychiatry 2005 62 593 602 10.1001/archpsyc.62.6.593 15939837

[B2] Bentley SD Chater KF Cerdeno-Tarraga AM Challis GL Thomson NR James KD Harris DE Quail MA Kieser H Harper D Bateman A Brown S Chandra G Chen CW Collins M Cronin A Fraser A Goble A Hidalgo J Hornsby T Howarth S Huang CH Kieser T Larke L Murphy L Oliver K O’Neil S Rabbinowitsch E Rajandream MA Rutherford K Complete genome sequence of the model actinomycete Streptomyces coelicolor A3(2) Nature 2002 417 141 147 10.1038/417141a 12000953

[B3] Simon GE Korff M Barlow W Health care costs of primary care patients with recognized depression Arch Gen Psychiatry 1995 52 850 856 10.1001/archpsyc.1995.03950220060012 7575105

[B4] Fergusson DM Horwood LJ Ridder EM Beautrais AL Subthreshold depression in adolescence and mental health outcomes in adulthood Arch Gen Psychiatry 2005 62 66 72 10.1001/archpsyc.62.1.66 15630074

[B5] Pine DS Cohen P Gurley D Brook J Ma Y The risk for early-adulthood anxiety and depressive disorders in adolescents with anxiety and depressive disorders Arch Gen Psychiatry 1998 55 56 64 10.1001/archpsyc.55.1.56 9435761

[B6] Burcusa SL Iacono WG Risk of recurrence in depression Clin Psychol Rev 2007 27 959 985 10.1016/j.cpr.2007.02.005 17448579 PMC2169519

[B7] Mueller TI Leon AC Keller MB Solomon DA Endicott J Coryell W Warshaw M Maser JD Recurrence after recovery from major depressive disorder during 15 years of observational follow-up Am J Psychiatry 1999 156 1000 1006 10401442 10.1176/ajp.156.7.1000

[B8] Anda R Williamson D Jones D Macera C Eaker E Glassman A Marks J Depressed affect, hopelessness, and the risk of ischemic disease in a cohort of U.S. adults Epidemiology 1993 4 285 294 10.1097/00001648-199307000-00003 8347738

[B9] Pan A Sun Q Okereke OL Rexrode KM Hu FB Depression and risk of stroke morbidity and mortality: a meta-analysis and systematic review JAMA 2011 306 1241 1249 10.1001/jama.2011.1282 21934057 PMC3242806

[B10] Golden SH Williams JE Ford DE Yeh HC Paton Sanford C Nieto FJ Brancati FL Atherosclerosis risk in communities study: depressive symptoms and the risk of type 2 diabetes: the atherosclerosis risk in communities study Diab Care 2004 27 429 435 10.2337/diacare.27.2.429 14747224

[B11] Smith JP Smith GC Long-term economic costs of psychological problems during childhood Soc Sci Med 2010 71 110 115 10.1016/j.socscimed.2010.02.046 20427110 PMC2887689

[B12] Craighead WE Sheets ES Brosse AL Ilardi SS Nathan PE, Gorman JM Psychosocial treatments for major depressive disorder A Guide to Treatments that Work 2007 3 New York: Oxford University Press 289 307

[B13] Nemeroff CB Schatzberg AF Nathan PE, Gorman JM Pharmacological treatments for unipolar depression A Guide to Treatments that Work 2007 3 New York: Oxford University Press 271 287

[B14] Frank E Prien RF Jarrett RB Keller MB Kupfer DJ Lavori PW Rush AJ Weissman MM Conceptualization and rationale for consensus definitions of terms in major depressive disorder: remission, recovery, relapse, and recurrence Arch Gen Psychiatry 1991 48 851 855 10.1001/archpsyc.1991.01810330075011 1929776

[B15] Trivedi MH Rush AJ Wisniewski SR Nierenberg AA Warden D Ritz L Norquist G Howland RH Lebowitz B McGrath PJ Shores-Wilson K Biggs MM Balasubramani GK Fava M STAR*D Study Team Evaluation of outcomes with citalopram for depression using measurement-based care in STAR*D: implications for clinical practice Am J Psychiatry 2006 163 28 40 10.1176/appi.ajp.163.1.28 16390886

[B16] Nierenberg AA Husain MM Trivedi MH Fava M Warden D Wisniewski SR Miyahara S Rush AJ Residual symptoms after remission of major depressive disorder with citalopram and risk of relapse: a STAR*D report Psychol Med 2010 40 41 50 10.1017/S0033291709006011 19460188 PMC5886713

[B17] Judd LL Akiskal HS Maser JD Zeller PJ Endicott J Coryell W Paulus MP Kunovac JL Leon AC Mueller TI Rice JA Keller MB A prospective 12-year study of subsyndromal and syndromal depressive symptoms in unipolar major depressive disorders Arch Gen Psychiatry 1998 55 694 700 10.1001/archpsyc.55.8.694 9707379

[B18] Simon GE Perlis RH Personalized medicine for depression: can we match patients with treatments? Am J Psychiatry 2010 167 1445 1455 10.1176/appi.ajp.2010.09111680 20843873 PMC3723328

[B19] Nemeroff CB Heim CM Thase ME Klein DN Schatzberg AF Ninan PT McCullough JP Weiss PM Dunner DL Rothbaum BO Kornstein S Keitner G Keller MB Differential responses to psychotherapy versus pharmacotherapy in patients with chronic forms of major depression and childhood trauma Proc Natl Acad Sci USA 2003 100 14293 14296 10.1073/pnas.2336126100 14615578 PMC283585

[B20] National Committee for Quality Assurance The State of Health Care Quality 2007 2007 Washington, DC: National Committee for Quality Assurance 20 21

[B21] McGrath P Stewart J Harrison W Ocepek-Welikson K Rabkin JG Nunes EN Wager SB Tricamo E Quitkin FM Klein DF Predictive value of symptoms of atypical depression for differential drug treatment outcome J Clin Psychopharmacol 1992 12 197 202 1629387

[B22] Fava M Rush AJ Alper JE Balasubramani GK Wisniewski SR Carmin CN Biggs MM Zisook S Leuchter A Howland R Warden D Trivedi MH Difference in treatment outcome in outpatients with anxious versus non-anxious depression: a STAR*D report Am J Psychiatry 2008 165 342 351 10.1176/appi.ajp.2007.06111868 18172020

[B23] Dunlop BW Aaron MH Response to treatment with placebo, medication or psychotherapy in severe non-psychotic major depressive disorder Curr Psychiatry Rev 2010 6 28 45 10.2174/157340010790596526

[B24] Trivedi M Morris DW Grannemann BD Mahadi S Symptom clusters as predictors of late response to antidepressant treatment J Clin Psychiatry 2005 66 1064 1070 10.4088/JCP.v66n0816 16086624

[B25] Kocsis JH Leon AC Markowitz JC Manber R Arnow B Klein DN Thase ME Patient preference as a moderator of outcome for chronic forms of major depressive disorder treated with nefazodone, cognitive behavioral analysis system of psychotherapy, or their combination J Clin Psychiatry 2009 70 354 361 10.4088/JCP.08m04371 19192474

[B26] Newton-Howes G Tyrer P Johnson T Personality disorder and the outcome of depression: meta-analysis of published studies Br J Psychiatry 2006 188 13 20 10.1192/bjp.188.1.13 16388064

[B27] Hunter AM Cook IA Leuchter AF Impact of antidepressant treatment history on clinical outcomes in placebo and medication treatment of major depression J Clin Psychopharmacol 2010 30 748 750 10.1097/JCP.0b013e3181faa474 21057245

[B28] Taylor BP Bruder GE Stewart JW McGrath PJ Halperin J Ehrlichman H Quitkin FM Psychomotor slowing as a predictor of fluoxetine nonresponse in depressed outpatients Am J Psychiatry 2006 163 73 78 10.1176/appi.ajp.163.1.73 16390892

[B29] DeRubeis RJ Hollon SD Amsterdam JD Shelton RC Young PR Salomon RM O‘Reardon JP Lovett ML Gladis MM Brown LL Gallop R Cognitive therapy vs medications in the treatment of moderate to severe depression Arch Gen Psychiatry 2005 62 409 416 10.1001/archpsyc.62.4.409 15809408

[B30] Juckel G Pogarell O Augustin H Mulert C Muller-Siecheneder F Frodl T Mavrogiorgou P Hegerl U Differential prediction of first clinical response to serotonergic and noradrenergic antidepressants using the loudness dependence of auditory evoked potentials in patients with major depressive disorder J Clin Psychiatry 2007 68 1206 1212 10.4088/JCP.v68n0806 17854244

[B31] Bruder GE Stewart JW Tenke CE McGrath PJ Leite P Bhattacharya N Quitkin FM Electroencephalographic and perceptual asymmetry differences between responders and nonresponders to an SSRI antidepressant Biol Psychiatry 2001 49 416 425 10.1016/S0006-3223(00)01016-7 11274653

[B32] Leuchter AF Cook IA Gilmer WS Marangell LB Burgoyne KS Howland RH Trivedi MH Zisook S Jain R Fava M Iosifescu D Greenwald S Effectiveness of a quantitative electroencephalographic biomarker for predicting differential response or remission with escitalopram and bupropion in major depressive disorder Psychiatry Res 2009 169 124 131 10.1016/j.psychres.2009.06.004 19709754

[B33] Ising M Horstmann S Kloiber S Lucae S Binder EB Kern N Kunzel HE Pfennig A Uhr M Holsboer F Combined dexamethasone/corticotropin releasing hormone test predicts treatment response in major depression - a potential biomarker? Biol Psychiatry 2007 62 47 54 10.1016/j.biopsych.2006.07.039 17123470

[B34] Maas JW Fawcett JA Dekirmenjian H Catecholamine metabolism, depressive illness, and drug response Arch Gen Psychiatry 1972 26 252 262 10.1001/archpsyc.1972.01750210060012 4551050

[B35] Kusumi I Suzuki K Sasaki Y Kameda K Koyama T Treatment response in depressed patients with enhanced Ca mobilization stimulated by serotonin Neuropsychopharmacol 2000 23 690 696 10.1016/S0893-133X(00)00149-4 11063924

[B36] Mayberg HS Modulating dysfunctional limbic-cortical circuits in depression: towards development of brain-based algorithms for diagnosis and optimised treatment Br Med Bull 2003 65 193 207 10.1093/bmb/65.1.193 12697626

[B37] MacQueen GM Magnetic resonance imaging and prediction of outcome in patients with major depressive disorder Can J Psychiatry 2009 34 343 349 PMC2732740 19721844

[B38] Konarski JZ Kennedy SH Segal ZV Lau MA Bieling PJ McIntyre RS Mayberg HS Predictors of non-response to cognitive behavioural therapy or venlafaxine using glucose metabolism in major depressive disorder J Psychiatry Neurosci 2009 34 175 180 19448846 PMC2674969

[B39] Horstmann S Binder EB Pharmacogenomics of antidepressant drugs Pharmacol Ther 2009 124 57 73 10.1016/j.pharmthera.2009.06.007 19563827

[B40] Kirchheiner J Brosen K Dahl ML Gram LF Kasper S Roots I Sjogvist F Spina E Brockmoller J CYP2D6 and CYP2C19 genotype-based dose recommendations for antidepressants: a first step towards subpopulationspecific dosages Acta Psychiatr Scand 2001 104 173 192 10.1034/j.1600-0447.2001.00299.x 11531654

[B41] Laje G McMahon FJ Genome-wide association studies of antidepressant outcome: a brief review Prog Neuropsychopharmacol Biol Psychiatry 2011 35 1553 1557 10.1016/j.pnpbp.2010.11.031 21115088 PMC3125482

[B42] Ising M Lucae S Binder E Bettecken T Uhr M Ripke S Kohli MA Hennings JM Hortsmann S Kloiber S Menke A Bondy B Rupprecht R Domschke K Baune BT Arolt V Rush AJ Holsboer F Muller-Myhsok B A genome-wide association study points to multiple loci predicting treatment outcome in depression Arch Gen Psychiatry 2009 66 966 975 10.1001/archgenpsychiatry.2009.95 19736353 PMC4465570

[B43] Uher R Perroud N Ng MY Hauser J Henigsberg N Maier W Mors O Placentino A Rietschel M Souery D Zagar T Czerski PM Jerman B Larsen ER Schulze TG Zobel A Cohen-Woods S Pirlo K Butler AW Muglia P Barnes MR Lathrop M Farmer A Breen G Aitchison KJ Craig I Lewis CM McGuffin P Genome-wide pharmacogenetics of antidepressant response in the GENDEP project Am J Psychiatry 2010 167 555 564 10.1176/appi.ajp.2009.09070932 20360315

[B44] National Institute of Mental Health National Institute of Mental Health Strategic Plan 2008 http://www.nimh.nih.gov/about/strategic-planning-reports/nimh-strategic-plan-2008.pdf

[B45] U.S. Census Bureau Percent of the Projected Population by Race and Hispanic Origin for the United States: 2010 to 2050 2008 NP2008-T6 http://www.census.gov/population/www/projections/summarytables.html

[B46] World Medical Association World Medical Association Declaration of Helsinki Ethical Principles for Medical Research Involving Human Subjects Last revised 2008 Oct http://www.wma.net/e/policy/b3.htm 10.1191/0969733002ne486xx 16010903

[B47] First MB Spitzer RL Gibbon M Williams JB Structured Clinical Interview for DSM-IV Axis I Disorders-Patient Edition (SCID-I/P, Version 2.0) 1995 New York: Biometrics Research Department, New York State Psychiatric Institute

[B48] Concepcion G De la Cruz A Structured Clinical Interview for DSM-IV-TR Axis I Disorders, Research Version, Patient Edition, (SCID-I/P), Spanish Translation 2005 New York: Biometrics Research: New York State Psychiatric Institute

[B49] Hamilton M Development of a rating scale for primary depressive illness Br J Soc Clin Psychol 1967 6 278 296 10.1111/j.2044-8260.1967.tb00530.x 6080235

[B50] Williams JB Standardizing the hamilton depression rating scale: past, present, and future Eur Arch Psychiatry Clin Neurosci 2001 251 Suppl 2 6 12 11824839 10.1007/BF03035120

[B51] Williams JB A structured interview guide for the Hamilton Depression Rating Arch Gen Psychiatry 1989 45 742 747 Spanish translation, Jan 2009, obtained from author 3395203 10.1001/archpsyc.1988.01800320058007

[B52] Montgomery SA Åsberg M A new depression scale designed to be sensitive to change Br J Psychiatry 1979 134 382 389 10.1192/bjp.134.4.382 444788

[B53] Williams JBW Kobak KA Development and Reliability of the SIGMA: A structured interview guide for the Montgomery-Asberg Depression Rating Scale (MADRS) Br J Psychiatry 2008 192 52 58 Spanish translation, Jan 2009, obtained from author 10.1192/bjp.bp.106.032532 18174510

[B54] Hamilton M The assessment of anxiety states by rating Br J Med Psychol 1959 32 50 55 10.1111/j.2044-8341.1959.tb00467.x 13638508

[B55] Guy W Clinical global impressions ECDEU Assessment Manual forPsychopharmacology, Revised, Volume 57 1976 Bethesda, MD: US Department of Health,Education and Welfare, National Institute of Mental Health 217 222

[B56] Bernstein DP Ahluvalia T Pogge D Handelsman L Validity of the Childhood Trauma Questionnaire in an adolescent psychiatric population J Am Acad Child Adolesc Psychiatry 1997 36 340 348 10.1097/00004583-199703000-00012 9055514

[B57] Lizardi H Klein DN Ouimette PC Riso LP Anderson RL Donaldson SK Reports of the childhood home environment in early-onset dysthymia and episodic major depression J Abnorm Psychol 1995 104 132 139 7897035 10.1037//0021-843x.104.1.132

[B58] Rush AJ Trivedi MH Ibrahim HM Carmody TJ Biggs MM Suppes T Crismon ML Shores-Wilson K Toprac MG Dennehy EB Witte B Kashner TM The 16-Item Quick Inventory of Depressive Symptomatology (QIDS), clinician rating (QIDS-C), and self-report (QIDS-SR): a psychometric evaluation in patients with chronic major depression Biol Psychiatry 2003 54 573 583 Spanish translation http://www.ids-qids.org/tr-spanish.html 10.1016/S0006-3223(02)01866-8 12946886

[B59] Beck AT Ward CH Mendelson M Mock J Erbaugh J An inventory for measuring depression Arch Gen Psychiatry 1961 4 561 571 10.1001/archpsyc.1961.01710120031004 13688369

[B60] Conde-López V Chamorro TE Useros-Serrano E Critical study of the reliability and validity of Beck’s Rating Scale for the measurement of depression Arch Neurobiol 1976 39 313 338 985005

[B61] Endicott J Nee J Harrison W Blumenthal R Quality of Life Enjoyment and Satisfaction Questionnaire: a new measure Psychopharmacol Bull 1993 29 321 326 8290681

[B62] Sheehan DV Rush AJ, Pincus HA, First MB, Blacker D, Endicott J, Keith SJ, Phillips KA, Ryan ND, Smith GR, Tsuang MT, Widiger TA, Zarin DA Sheehan Disability Scale Handbook of Psychiatric Measures 2000 Washington, DC: American Psychiatric Association 113 115

[B63] Bobes J Badia X Luque A Garcia M Gonzalez MP Dal-Re R Validation of the Spanish versions of the Liebowitz Social Anxiety Scale, Social Anxiety and Distress Scale and Sheehan Disability Inventory for the evaluation of social phobia Med Clin (Barc) 1999 112 530 538 10363239

[B64] Dunlop BW Kelley ME Mletzko TC Velasquez CM Craighead WE Mayberg HS Depression beliefs, treatment preference, and outcomes in a randomized trial for major depressive disorder J Psychiatr Res 2012 46 375 381 10.1016/j.jpsychires.2011.11.003 22118808 PMC3288535

[B65] Sarason I Johnson J Siegel J Assessing the impact of life changes: development of the Life Experiences Survey J Consult Clin Psychol 1978 46 932 946 701572 10.1037//0022-006x.46.5.932

[B66] Papakostas GI Thase ME Fava M Nelson JC Shelton RC Are antidepressant drugs that combine serotonergic and noradrenergic mechanisms of action more effective than the selective serotonin reuptake inhibitors in treating major depressive disorder? A meta-analysis of studies of newer agents Biol Psychiatry 2007 62 1217 1227 10.1016/j.biopsych.2007.03.027 17588546

[B67] Nemeroff CB Entsuah R Benattia I Demitrack M Sloan DM Thase ME Comprehensive Analysis of Remission (COMPARE) with Venlafaxine versus SSRIs Biol Psychiatry 2008 63 424 434 10.1016/j.biopsych.2007.06.027 17888885

[B68] Thase ME Pritchett YL Ossanna MJ Swindle RW Xu J Detke MJ Efficacy of duloxetine and selective serotonin reuptake inhibitors: comparisons as assessed by remission rates in patients with major depressive disorder J Clin Psychopharmacol 2007 27 672 676 10.1097/jcp.0b013e31815a4412 18004135

[B69] Kennedy SH Andersen HF Lam RW Efficacy of escitalopram in the treatment of major depressive disorder compared with conventional selective serotonin reuptake inhibitors and venlafaxine XR: a meta-analysis J Psychiatry Neurosci 2006 31 122 131 16575428 PMC1413963

[B70] Cipriani A Furukawa TA Salanti G Geddes JR Higgins JP Churchill R Watanabe N Nakagawa A Omori IM McGuire H Tansella M Barbui C Comparative efficacy and acceptability of 12 new-generation antidepressants: a multiple-treatments meta-analysis Lancet 2009 373 746 758 10.1016/S0140-6736(09)60046-5 19185342

[B71] Ritchie JC Glover B Ramsey C Scott-Harrell P A routine UPLC-MS//MS assay for the newer antidepressants Ther Drug Monit 2009 31 646

[B72] Hostetter AL Stowe ZN Cox M Ritchie JC A novel system for the determination of antidepressants in human breast milk Ther Drug Monit 2004 26 47 52 10.1097/00007691-200402000-00011 14749550

[B73] Beck AT Rush AJ Shaw BF Emery G Cognitive Therapy of Depression 1979 New York, NY: Guilford

[B74] Feeley M DeRubeis RJ Gelfand LA The temporal relation of adherence and alliance to symptom change in cognitive therapy for depression J Consult Clin Psychol 1999 67 578 582 10450629 10.1037//0022-006x.67.4.578

[B75] Fawcett J Epstein P Fiester SJ Elkin I Autry JH Clinical management-imipramine/placebo administration manual: NIMH Treatment of Depression Collaborative Research Program Psychopharm Bull 1987 23 309 324 3303100

[B76] Keller MB Lavori PW Friedman B Nielsen E Endicott J McDonald-Scott P Andreasen NC The longitudinal interval follow-up evaluation. A comprehensive method for assessing outcome in prospective longitudinal studies Arch Gen Psychiatry 1987 44 540 548 10.1001/archpsyc.1987.01800180050009 3579500

[B77] Greicius MD Krasnow B Reiss AL Menon V Functional connectivity of the resting brain: a network analysis of the default mode hypothesis Proc Nat Acad Sci USA 2003 100 253 258 10.1073/pnas.0135058100 12506194 PMC140943

[B78] Peltier SJ Polk TA Noll DC Detecting low-frequency functional connectivity in fMRI using a self-organizing map (SOM) Algorithm Human Brain Mapping 2003 20 220 226 10.1002/hbm.10144 PMC6872081 14673805

[B79] Horwitz B Friston KJ Taylor JG Neural modeling and functional brain imaging: an overview Neural Netw 2000 13 829 846 10.1016/S0893-6080(00)00062-9 11156195

[B80] Greicius MD Flore BH Menon V Glover GH Solvason HB Kenna H Reiss AL Schatzberg AF Resting-state functional connectivity in major depression: abnormally increased contributions from subgenual cingulate cortex and thalamus Biol Psychiatry 2007 62 429 437 10.1016/j.biopsych.2006.09.020 17210143 PMC2001244

[B81] Anand A Li Y Wang Y Wu J Gao S Bukhari L Mathews VP Kalnin A Lowe MJ Activity and connectivity of brain mood regulating circuit in depression: a functional magnetic resonance study Biol Psychiatry 2005 57 1079 1088 10.1016/j.biopsych.2005.02.021 15866546

[B82] Craddock RC Holtzheimer PE Hu XP Mayberg HS Disease state prediction from resting state functional connectivity Mag Resonance Med 2009 62 1619 1628 10.1002/mrm.22159 PMC3749911 19859933

[B83] Drevets WC Price JL Furey ML Brain structure and functional abnormalities in mood disorders: implications for neurocircuitry models of depression Brain Struct Funct 2008 213 93 118 10.1007/s00429-008-0189-x 18704495 PMC2522333

[B84] Beckmann CF DeLuca M Devlin JT Smith SM Investigations into resting-state connectivity using independent component analysis Phil Trans R Soc B 2005 360 1001 1013 10.1098/rstb.2005.1634 16087444 PMC1854918

[B85] Ashburner J Friston KJ Voxel-based morphometry - the methods Neuroimage 2000 11 806 821 10.1016/S1053-8119(00)91735-X 10860804

[B86] Johansen-Berg H Gutman D Behrens T Matthews P Rushworth M Katz E Lozano A Mayberg HS Anatomical connectivity of subgenual cingulate region targeted with DBS for treatment resistant depression Cereb Cortex 2008 18 1374 1383 10.1093/cercor/bhm167 17928332 PMC7610815

[B87] James GA Kelley ME Craddock RC Holtzheimer PE Dunlop B Nemeroff C Hu XP Mayberg HS Exploratory structural equation modeling of resting-state fMRI: applicability of group models to individual subjects Neuroimage 2009 45 778 787 10.1016/j.neuroimage.2008.12.049 19162206 PMC2653594

[B88] Clark LA Watson D Mood and the mundane: relations between daily life events and self-reported mood J Pers Soc Psychol 1988 54 296 308 3346815 10.1037//0022-3514.54.2.296

[B89] Keers R Aitchison KJ Pharmacogenetics of antidepressant response Expert Rev Neurother 2011 11 101 125 10.1586/ern.10.186 21158559

[B90] Baer-Dubowska W Majchrzak-Celińska A Cichocki M Pharmocoepigenetics: a new approach to predicting individual drug responses and targeting new drugs Pharmacol Rep 2011 63 293 304 21602587 10.1016/s1734-1140(11)70498-4

[B91] Menke A Arloth J Pütz B Weber P Klengel T Mehta D Gonik M Rex-Haffner M Rubel J Uhr M Lucae S Deussing JM Müller-Myhsok B Holsboer F Binder EB Dexamethasone stimulated gene expression in peripheral blood is a sensitive marker for glucocorticoid receptor resistance in depressed patients Neuropsychopharmacology 2012 37 1455 1464 10.1038/npp.2011.331 22237309 PMC3327850

[B92] Mehta D Menke A Binder EB Gene expression studies in major depression Curr Psychiatry Rep 2010 12 135 144 10.1007/s11920-010-0100-3 20425299 PMC2847693

[B93] Holsboer F von Bardeleben U Wiedemann K Muller OA Stalla GK Serial assessment of corticotropin releasing hormone response after dexamethasone in depression: implications of pathophysiology of DST suppression Biol Psychiatry 1987 22 228 234 10.1016/0006-3223(87)90237-X 3028512

[B94] Ising M Künzel HE Binder EB Nickel T Modell S Holsboer F The combined dexamethasone/CRH test as a potential surrogate marker in depression Prog Neuropsychopharm Biol Psychiatry 2005 29 1085 1093 10.1016/j.pnpbp.2005.03.014 15950349

[B95] Heuser I Yassouridis A Holsboer F The combined dexamethasone/CRH test: a refined laboratory test for psychiatric disorders J Psychiatr Res 1994 28 341 356 10.1016/0022-3956(94)90017-5 7877114

[B96] Holsboer F The corticosteroid receptor hypothesis of depression Neuropsychopharmacol 2000 23 477 501 10.1016/S0893-133X(00)00159-7 11027914

[B97] Hatzinger M Hemmeter UM Baumann K Brand S Holsboer-Trachsler E The combined DEX/CRH test in treatment course and long-term outcome of major depression J Psychiatric Res 2002 36 287 297 10.1016/S0022-3956(02)00021-3 12127596

[B98] Kunugi H Ida I Owashi T Kimura M Inoue Y Nakagawa S Yabana T Urushibara T Kanai R Aihara M Yuuki N Otsubo T Oshima A Kuo K Inoue T Kitaichi Y Shirakawa O Isogawa K Nagayama H Kamijima K Nanko S Kanba S Higuchi T Mikuni M Assessment of the dexamethasone/CRH test as a state-dependent marker for hypothalamic-pituitary-adrenal (HPA) axis abnormalities in major depressive episode: a multicenter study Neuropsychopharmacol 2006 31 212 220 10.1038/sj.npp.1300868 16123748

[B99] Binder EB Kunzel HE Nickel T Kern N Pfennig A Majer M Uhr M Ising M Holsboer F HPA-axis regulation at in-patient admission is associated with antidepressant therapy outcome in male but not in female depressed patients Psychoneuroendocrinol 2009 34 99 109 10.1016/j.psyneuen.2008.08.018 18829172

[B100] Aubry JM Gervasoni N Osiek C Perret G Rossier MF Bertschy G Bondolfi G The DEX/CRH neuroendocrine test and the prediction of depressive relapse in remitted depressed outpatients J Psychiatric Res 2007 41 290 294 10.1016/j.jpsychires.2006.07.007 16956623

[B101] Dunlop BW Betancourt Y Binder EB Heim C Holsboer F Ising M McKenzie M Mletzko T Pfister H Nemeroff CB Craighead WE Mayberg HS Tolerability of the dexamethasone-corticotropin eleasing hormone test in major depressive disorder J Psychiatric Res 2011 45 24 28 10.1016/j.jpsychires.2010.04.020 PMC2950910 20488460

[B102] Heim C Bradley B Mletzko TC Deveau TC Musselman DL Nemeroff CB Ressler KJ Binder EB Effect of childhood trauma on adult depression and neuroendocrine function: sex-specific moderation by CRH receptor 1 gene Front Behav Neurosci 2009 3 41 20161813 10.3389/neuro.08.041.2009 PMC2821197

[B103] Howren MB Lamkin DM Suls J Associations of depression with C-reactive protein, IL-1, and IL-6: a meta-analysis Psychosom Med 2009 71 171 186 10.1097/PSY.0b013e3181907c1b 19188531

[B104] Dowlati Y Herrmann N Swardfager W Liu H Sham L Reim EK Lanctot KL A meta-analysis of cytokines in major depression Biol Psychiatry 2011 67 446 457 20015486 10.1016/j.biopsych.2009.09.033

[B105] Liu Y Ho RC Mak A Interleukin (IL)-6, tumour necrosis factor alpha (TNF-alpha) and soluble interleukin-2 receptors (sIL-2R) are elevated in patients with major depressive disorder: A meta-analysis and meta-regression J Affect Disord 2011 139 230 239 21872339 10.1016/j.jad.2011.08.003

[B106] Alesci S Martinez PE Kelkar S Ilias I Ronsaville DS Listwak SJ Licinio J Gold HK Kling MA Chrousos GP Gold PW Major depression is associated with significant diurnal elevations in plasma interleukin-6 levels, a shift of its circadian rhythm, and loss of physiological complexity in its secretion: clinical implications J Clin Endocr Metab 2005 90 2522 2530 10.1210/jc.2004-1667 15705924

[B107] Elovainio M Aalto AM Kivimaki M Pirkola S Sundvall J Lonnqvist J Reunanen A Depression and C-reactive protein: population-based Health 2000 Study Psychosom Med 2009 71 423 430 10.1097/PSY.0b013e31819e333a 19297307

[B108] Bremmer MA Beekman AT Deeg DJ Penninx BW Dik MG Hack CE Hoogendijk WJ Inflammatory markers in late-life depression: results from a population-based study J Affect Disord 2008 106 249 255 10.1016/j.jad.2007.07.002 17716746

[B109] Suarez EC C-reactive protein is associated with psychological risk factors of cardiovascular disease in apparently healthy adults Psychosom Med 2004 66 684 691 10.1097/01.psy.0000138281.73634.67 15385692

[B110] Musselman DL Lawson DH Gumnick JF Manatunga AK Penna S Goodkin RS Greiner K Nemeroff CB Miller AH Paroxetine for the prevention of depression induced by high-dose interferon alfa N Eng J Med 2001 344 961 966 10.1056/NEJM200103293441303 11274622

[B111] Turnbull AV Rivier CL Regulation of the hypothalamic-pituitary-adrenal axis by cytokines: actions and mechanisms of action Physiological Rev 1999 79 1 71 10.1152/physrev.1999.79.1.1 9922367

[B112] Pace TW Miller AH Cytokines and glucocorticoid receptor signaling. Relevance to major depression Ann N Y Acad Sci 2009 1179 86 105 10.1111/j.1749-6632.2009.04984.x 19906234 PMC3399249

[B113] Irwin MR Miller AH Depressive disorders and immunity: 20 years of progress and discovery Brain Behav Immun 2007 21 374 383 10.1016/j.bbi.2007.01.010 17360153

[B114] Raison CL Capuron L Miller AH Cytokines sing the blues: inflammation and the pathogenesis of major depression Trends Immunol 2006 27 24 31 10.1016/j.it.2005.11.006 16316783 PMC3392963

[B115] R&D Systems Quantikine® HS ELISA Human IL-6 Immunoassay 2012 http://www.mdsystems.com/pdf/hs600b.pdf

[B116] Pace TWW Negi LT Adame DD Cole SP Sivilli TI Brown TD Issa MJ Raison CL Effect of compassion meditation on neuroendocrine, innate immune and behavioral responses to psychosocial stress Psychoneuroendocrinology 2009 34 87 98 10.1016/j.psyneuen.2008.08.011 18835662 PMC2695992

[B117] Craighead WE Sheets ES Craighead LW Madsen JW Recurrence of MDD: A prospective study of personality pathology and cognitive distortions Personality Disorders: Theory, Research, and Treatment 2011 2 83 97 10.1037/a0020456 22448730

[B118] Skodol AE Grilho CM Keyes KM Geier T Grant BF Hasin DS Relationship of personality disorders to the course of major depressive disorder in a nationally representative sample Am J Psychiatry 2011 168 257 264 10.1176/appi.ajp.2010.10050695 21245088 PMC3202962

[B119] Ilardi SS Craighead WE Evans DD Modeling relapse in unipolar depression: the effects of dysfunctional cognitions and personality disorders J Consult Clin Psychol 1997 65 381 391 9170761 10.1037//0022-006x.65.3.381

[B120] Westen D Shedler J Bradley B DeFife JA An empirically derived taxonomy for personality diagnosis: bridging science and practice in conceptualizing personality Am J Psychiatry 2012 169 273 284 10.1176/appi.ajp.2011.11020274 22193534 PMC4546840

[B121] Bagby RM Quilty LC Segal ZV McBride CC Kennedy SH Costa PT Personality and differential treatment response in major depression: A randomized controlled trial comparing cognitive-behavioural therapy and pharmacotherapy Can J Psychiatry 2008 53 361 370 18616856 10.1177/070674370805300605 PMC2543930

[B122] Westen D Waller N Shedler J Blagov P Dimensions of personality and personality pathology: Factor structure of the Shedler-Westen Assessment Procedure-II (SWAP-II) J Pers Disordin press 10.1521/pedi_2012_26_059 22984863

[B123] Westen D Shedler J Personality diagnosis with the Shedler-Westen Assessment Procedure (SWAP): Integrating clinical and statistical measurement and prediction J Abnorm Psychol 2007 116 810 822 18020727 10.1037/0021-843X.116.4.810

[B124] Loranger AW Sartorius N Andreoli A Berger P Buchheim P Channabasavanna SM Coid B Dahl A Diekstra RF Ferguson B Jacobsberg LB Mombour W Pull C Ono Y Regier DA The international personality disorder examination Arch Gen Psychiatry 1994 51 215 224 10.1001/archpsyc.1994.03950030051005 8122958

[B125] Snyder CR Harris JF Anderson SA Holleran SA Irving LM Sigmon ST Yoshinobu L Gibb J Langelle C Harney P The will and the ways: development and validation of an individual differences measure of hope J Pers Soc Psychol 1991 60 570 585 2037968 10.1037//0022-3514.60.4.570

[B126] DeLongis A Folkman S Lazarus R The impact of daily stress on health and mood: psychological social resources as mediators J Pers Soc Psychol 1988 54 486 495 3361420 10.1037//0022-3514.54.3.486

[B127] Scarnell RW Umanath NS Data Modeling and Database Design 2007 Boston, MA: Thomson Course Technology

[B128] National Institutes of Health Research Repositories, Databases, and the HIPAA Privacy Rule. NIH Publication Number 04–5489 2004 http://privacyruleandresearch.nih.gov/research_repositories.asp

[B129] Antman EM Cohen M Bernink PJ McCabe CH Horacek T Papuchis G Mautner B Corbalan R Radley D Braunwald E The TIMI risk score for unstable angina/non-ST elevation MI: A method for prognostication and therapeutic decision making JAMA 2000 284 835 842 10.1001/jama.284.7.835 10938172

[B130] Breiman L Friedman JH Olshen RA Stone CJ Classification and regression trees 1984 Monterey, CA: Wadsworth

[B131] Tibshirani R Regression shrinkage and selection via the lasso J Royal Statistical Soc Series B 1996 58 267 288

[B132] Harrell FE Lee KL Mark DB Multivariable prognostic models: Issues in developing models, evaluating assumptions and adequacy, and measuring and reducing errors Statist Med 1996 15 361 387 10.1002/(SICI)1097-0258(19960229)15:4<361::AID-SIM168>3.0.CO;2-4 8668867

[B133] Pencina MJ D‘Agostino RB D’Agostino RB Vasan RS Evaluating the added predictive ability of a new marker: From area under the ROC curve to reclassification and beyond Statist Med 2008 27 157 172 10.1002/sim.2929 17569110

[B134] Sullivan LM Massaro JM D’Agostino RB Presentation of multivariate data for clinical use: The Framingham Study risk score functions Statist Med 2004 23 1631 1660 10.1002/sim.1742 15122742

[B135] Nierenberg AA Predictors of response to antidepressants: general principles and clinical implications Psychiatric Clin N Am 2003 26 345 352 10.1016/S0193-953X(02)00105-3 12778837

[B136] Cohen J Statistical power analysis for the behavioral sciences 1988 2 Hillsdale, NJ: Lawrence Earlbaum Associates

[B137] Bhagwagar Z Cohen PJ ‘It's not over when it's over’: persistent neurobiological abnormalities in recovered depressed patients Psychol Med 2008 38 307 313 18444278 10.1017/s0033291707001250

[B138] Papakostas GI Stahl SM Krishen A Seifert CA Tucker VL Goodale EP Fava M Efficacy of bupropion and the selective serotonin reuptake inhibitors in the treatment of major depressive disorder with high levels of anxiety (anxious depression): a pooled analysis of 10 studies J Clin Psychiatry 2008 69 1287 1292 10.4088/JCP.v69n0812 18605812

[B139] Owens MJ Knight DL Nemeroff CB Paroxetine binding to the rat norepinephrine transporter in vivo Biol Psychiatry 2000 47 842 845 10.1016/S0006-3223(99)00314-5 10812044

[B140] Greenblatt DJ von Moltke LL Harmatz JS Shader RI Drug interactions with newer antidepressants: role of human cytochromes P450 J Clin Psychiatry 1998 59 Suppl 15 19 27 9786307

[B141] Howell C Wilson AD Waring WS Cardiovascular toxicity due to venlafaxine poisoning in adults: a review of 235 consecutive cases Br J Pharmacol 2007 64 192 197 10.1111/j.1365-2125.2007.02849.x PMC2000637 17298480

[B142] Nemeroff CB Owens MJ Treatment of mood disorders Nat Neurosci 2002 5 Suppl 1068 1070 12403988 10.1038/nn943

[B143] Owens MJ Krulewicz S Simon JS Sheehan DV Thase ME Carpenter DJ Plott SJ Nemeroff CB Estimates of serotonin and norepinephrine transporter inhibition in depressed patients treated with paroxetine or venlafaxine Neuropsychopharmacol 2008 33 3201 3212 10.1038/npp.2008.47 18418363

[B144] Alexopolous GS Murphy CF Gunning-Dixon FM Latoussakis V Kanellopolous D Klimstra S Lim KO Hoptman MJ Microstructural white matter abnormalities and remission of geriatric depression Am J Psychiatry 2008 165 238 244 10.1176/appi.ajp.2007.07050744 18172016

